# Diverse effects of distance cutoff and residue interval on the performance of distance-dependent atom-pair potential in protein structure prediction

**DOI:** 10.1186/s12859-017-1983-3

**Published:** 2017-12-08

**Authors:** Yuangen Yao, Rong Gui, Quan Liu, Ming Yi, Haiyou Deng

**Affiliations:** 1 Department of Physics, College of Science, Huazhong Agricultural University, Wuhan, 430070 China; 2 Institute of Applied Physics, Huazhong Agricultural University, Wuhan, 430070 China

**Keywords:** Distance-dependent atom-pair potential, Protein structure prediction, Distance cutoff, Residue interval, Reference state

## Abstract

**Background:**

As one of the most successful knowledge-based energy functions, the distance-dependent atom-pair potential is widely used in all aspects of protein structure prediction, including conformational search, model refinement, and model assessment. During the last two decades, great efforts have been made to improve the reference state of the potential, while other factors that also strongly affect the performance of the potential have been relatively less investigated.

**Results:**

Based on different distance cutoffs (from 5 to 22 Å) and residue intervals (from 0 to 15) as well as six different reference states, we constructed a series of distance-dependent atom-pair potentials and tested them on several groups of structural decoy sets collected from diverse sources. A comprehensive investigation has been performed to clarify the effects of distance cutoff and residue interval on the potential’s performance. Our results provide a new perspective as well as a practical guidance for optimizing distance-dependent statistical potentials.

**Conclusions:**

The optimal distance cutoff and residue interval are highly related with the reference state that the potential is based on, the measurements of the potential’s performance, and the decoy sets that the potential is applied to. The performance of distance-dependent statistical potential can be significantly improved when the best statistical parameters for the specific application environment are adopted.

**Electronic supplementary material:**

The online version of this article (10.1186/s12859-017-1983-3) contains supplementary material, which is available to authorized users.

## Background

One of the major challenges in protein structure prediction is to design accurate energy function that can discriminate native or near-native structure from non-native structures [1]. Especially in conformational search [2–5], model refinement [6, 7] and model assessment [8–12], energy function is always the primary issue to be conquered. Although the detailed interactions of protein atoms can be described by quantum mechanical equations [13, 14], the amount of computation for such kind of macromolecule can easily go beyond the capability of current computing resources. The common practice is to approximate the interactions based on the classical physics [15]. These energy functions generally contain terms associated with bond lengths, bond angles, torsion angles, van der Waals interactions, and electrostatic interactions, which are often called physics-based energy function [16, 17]. By virtue of the abundant structure resources in Protein Data Bank [18], another category of energy function (called knowledge-based energy function [19, 20]) springs up and plays an increasingly important role in protein structure prediction. So far the most successful prediction methods are more or less based on the knowledge-based energy function [21–24].

Any aspect of structural features which characterize particular interactions in the folded proteins can be used to derive knowledge-based energy functions, especially those in pairwise form. The distance-dependent atom-pair potential [9, 25–29] is one of the most commonly used pairwise energy functions, which characterizes the distributions of pairwise distances between residue-specific atom types in protein structures, and converts them into energy based on the inverse of Boltzmann’s law. Many distance-dependent atom-pair potentials have been developed and widely used during the last two decades, such as RAPDF [25], KBP [26], Dfire [27], Dope [9], RW [29] and so on. Some potentials (e.g. dDFIRE [30], RWplus [29], GOAP [31], ROTAS [32]) also combine other energy terms for characterizing side-chain orientation, angle distribution, solvent accessibility or secondary structure preference, but the distance-dependent terms still play the central role. In order to develop more efficient distance-dependent atom-pair potential, great efforts have been made to improve the reference state, which makes the reference state the major difference between different potentials [33]. In fact, Many other factors also strongly affect the performance of distance-dependent atom-pair potential [34]. Distance cutoff (interactions of atom pairs with distances larger than the cutoff will be ignored) and residue interval (only atom pairs from two residues with sequential intervals equal or larger than the specified residue interval are considered) are two important statistical parameters for designing distance-dependent atom-pair potentials. RAPDF chooses a relatively large distance cutoff of 20 Å after testing four different values (5, 10, 15, and 20 Å) on the same decoy sets. KBP and Dfire set the distance cutoff to14.5 Å, whereas Dope and RW take distance cutoffs of 15 and 15.5 Å, respectively. Despite its importance, the distance cutoff was often determined without a careful optimization in many potentials. Similar to the situation of distance cutoff, the residue intervals in different potentials are usually set to different values, such as 1 (meaning that only atom-pairs within the same residue are excluded from the statistics), 5, 10 and so on. So far it is unclear what the optimal distance cutoff (or residue interval) is, and how it is related to the reference state and the decoy sets that the potential is applied to.

To specifically explore the effects of distance cutoff and residue interval on the performance of distance-dependent atom-pair potential, we constructed a series of potentials with different distance cutoffs and residue intervals as well as different reference states. All potentials were tested on several groups of structural decoy sets collected from diverse sources. We investigated the performance variations of these potentials in native recognition and decoy discrimination. We also explored the preferences of optimal distance cutoff and residue interval for different decoy sets and potentials with different reference states. The evaluation results have been compared with several widely used statistical potentials. Moreover, we applied the potentials with other residue intervals rather than used in potential construction, which yielded better performance in many cases. The results and observations of this work provide new insights and valuable references for determination of distance cutoff and residue interval to optimize the performance of distance-dependent atom-pair potential.

## Methods

### Distance-dependent atom-pair potentials with different reference states

The distance-dependent atom-pair potential is derived by counting the pair-wise distances of every two non-hydrogen atoms in protein structures. With the assumption that the distributions of structural features obtained from protein structures obey the Boltzmann distribution of statistical mechanics [19], the potential can be written as:

$$ {\overline{u}}_{i,j}(r)=-{k}_{\mathrm{B}}T\ln \left[\frac{f_{i,j}^{OBS}(r)}{f_{i,j}^{REF}(r)}\right] $$

where *k*_B_ and *T* are Boltzmann constant and Kelvin temperature, respectively.${f}_{i,j}^{OBS}(r)$ is the observed probability of atom types *i* and *j* in a particular distance bin *r* to *r+Δr* in native structures, which can be calculated from a non-redundant set of experimental structures. ${f}_{i,j}^{REF}(r)$ is the reference probability of atom types *i* and *j* in the corresponding distance bin in the non-native structures. Since such a structural database does not exist for non-native structures, how to deal with the reference state for calculating ${f}_{i,j}^{REF}(r)$ is a critical issue in designing potentials. We conducted our research on six well-known reference states. The basic information of these reference states are shown in Table 1 and more details can be found in our previous research article [33].

**Table 1:** Brief description of six reference states for distance-dependent atom-pair potential

Reference state^a^	Description
Averaging (ave-)	Take the average distance distribution over different atom types from experimental conformations as the reference state, which means the distance distributions for all types of atom pair are identical in the reference state [25].
Quasi-chemical approximation (kbp-)	Use the overall distance distribution of atom pair from experimental structures and calculate the specific distance distribution of atom types i and j based on the mole fraction (on the whole dataset) of atom type i and j [26].
Finite ideal-gas (dfire-)	Treat the reference state as finite ideal-gas that probability of atom pair in a particular distance bin increases in ra with a to-be-determined constant a (a < 2) [27].
Spherical non-interacting (dope-)	Treat the reference state as a sphere in which all atoms of a protein evenly distributed without ineraction. The size of sphere is specifically decided by corresponding experimental structure [9].
Random-walk chain (rw-)	Treat the reference state as an ideal random-walk chain of a rigid step length, which mimics well the generic entropic elasticity and inherent connectivity of polymer protein molecules and yet ignores the atomic interactions of amino acids [29].
Atom-shuffled (srs-)	Generate a shuffled structure dataset by preserving all atomic positions while shuffling atom identities within each of the experimental structures [28].

^a^The abbreviation is given in parentheses

### Potential construction with different distance cutoffs and residue intervals

We constructed a series of distance-dependent atom-pair potentials based on the aforementioned reference states with different distance cutoffs and residue intervals. A non-redundant structural dataset of 1762 proteins with pairwise sequence identity of <20%, resolution of <1.6 Å and R-factor of <0.25 was obtained from the PISCES webserver [35]. Proteins less than 50 residues or discontinuous in sequence in the original set were already discarded.

**Fig. 1:**
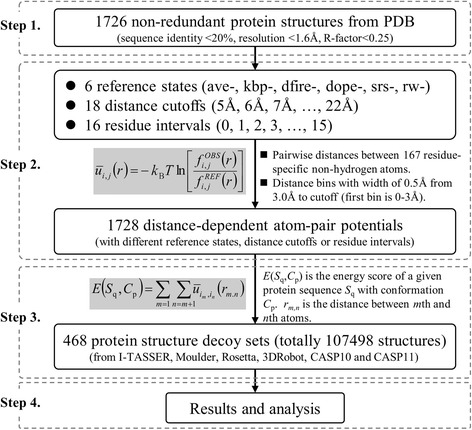
The flowchart of our studies. Step 1. PDB dataset preparation; Step 2. Potential construction; Step 3. Potential application; Step 4. Result analysis

All non-hydrogen atoms in each protein of the structural dataset have been considered for potential construction, and the description of the atoms is residue specific, for example, the Cα of lysine is different from the Cα of leucine. Thus, a total of 167 atom types have been defined. Since the amino acid sequence is asymmetric (with C and N terminal), the atom pair *i,j* and *j,i* were considered as different pairs and the total number of atom pairs is 27,889. The atom-pair distance is divided into different bins (0.5 Å in width) ranging from 3.0 Å to cutoff except for the first bin whose width is 3.0 Å. We implemented 18 distance cutoffs from 5.0 Å to 22.0 Å with the spacing of 1.0 Å, so the numbers of distance bins for potentials with different cutoffs ranged from 5 to 39. We also implemented 16 residue intervals from 0 to 15, where a residue interval of 0 means the atom pairs within one residue or in different residues with any sequential interval are all considered for potential construction. Eventually, we constructed 1728 (by 6 × 18 × 16) distance-dependent atom-pair potentials with different reference states, distance cutoffs or residue intervals. Figure 1 demonstrates the whole process from dataset preparation to result analysis.

To verify the statistical validity of distance distributions for all atom pairs, we checked the occurrence frequency of each atom pairs for several extreme cases. Potentials with distance cutoff of 5.0 Å and residue interval of 15 (abbreviated as P-5-15) are the ones most likely to encounter the sparse data problem. Additional file 1: Figure S1 shows that the minimum occurrence frequency for P-5-15 is 12 (from the atom pair of SER-OG and TRP-CA). Nearly 90% of atom pairs have more than 64 occurrences, which is sufficient for a potential with only 5 distance bins. Occurrence frequencies for potentials with higher distance cutoff and residue interval increase quickly (as shown in Additional file 1: Figure S1).

Moreover, the residue interval adopted in the potential application is not necessarily the same as that have been adopted for potential construction. To our surprise, we found that adopting different residue intervals in potential application and construction sometimes resulted in much better performance compared with adopting the same residue intervals. Therefore, in this article we tested all 16 residue intervals in every potential application no matter which residue interval have been adopted for potential construction. In this way, we can obtain 16 different energy scores when applying one potential to a protein structure.

### Protein structure decoy sets

We collected a large amount of protein structure decoy sets to evaluate the potentials we constructed. These decoy sets were generated by diverse methods and have different characteristics (as shown in Table 2), which composed a comprehensive environment for potential application. The I-TASSER decoy sets [29] contain 56 non-redundant proteins whose structure decoys (300–500 decoys for each protein) were generated by I-TASSER Monte Carlo simulations and refined by GROMACS4.0 MD simulation [36]. The Rosetta decoy sets [37] were generated by Rosetta ab initio structure prediction and each set includes 100 structure decoys (a total of 5858 structures for 58 proteins). The Moulder decoy sets [38] include 20 protein and their comparative models generated by the homology-modeling tool Modeller. The 3DRobot decoy sets were generated by the fragment assembly method we previously developed [39], which include 200 non-redundant proteins and a total of 60,200 structures. The CASP10 and CASP11 decoy sets were directly downloaded from http://predictioncenter.org. We removed the structures that are sequentially non-consecutive (the entire set will be removed if the experimental structure is non-consecutive in sequence) or shorter than the corresponding experimental structure. Furthermore, we trimmed all predicted structures to keep them identical in sequence to the experimental structure. The final decoy sets from CASP10 and CASP11 contain 72 proteins (a total of 5805 structures) and 62 proteins (a total of 4522 structures), respectively.

**Table 2:** Basic information of the six groups of structural decoy sets

Sets Name	Number of sets	Average length^a^	Number of structures
I-TASSER	56	80 (47–118)	24,707
Moulder	20	174 (81–340)	6406
Rosetta	58	83 (50–146)	5858
3DRobot	200	133 (80–240)	60,200
CASP10	72	224 (24–587)	5805
CASP11	62	206 (37–462)	4522
Total/Ave	468	146	107,498

^a^The length range is given in parentheses

### Performance measures

The performance of all potentials is evaluated by two categories of measurement. The first one (R1-num and Z-score) aimed to evaluate the ability of recognizing native (experimental) structure within a structural decoy set. R1-num refers to the number of decoy sets whose native structure is given the lowest energy score by the potential. Z-score is defined as (<*E*_*decoy*_> − *E*_native_)/*δ*, where *E*_native_is the energy score of the native structure, and <*E*_*decoy*_> and *δ*present the average score over all structural decoys and the standard deviation respectively. Therefore, the higher the Z-score is, the better is the ability of native recognition. The second category of measurement aimed to evaluate the ability of distinguishing near-native structures from non-native ones. In this paper, we calculated the Pearson’s correlation coefficient (PCC) between the energy score and TM-score [40] of all structures in the set, including the native structure.

## Results and discussion

### Overview of the performance variation of potentials

We constructed 1728 distance-dependent atom-pair potentials by different reference states, distance cutoffs and residue intervals, and applied them to 468 protein structure decoy sets collected from different sources. The results show that the choices of distance cutoff and residue interval have significant effects on the performance of the distance-dependent atom-pair potential. Here, we summarized the overall performances of these potentials in native recognition and decoy discrimination.

**Fig. 2:**
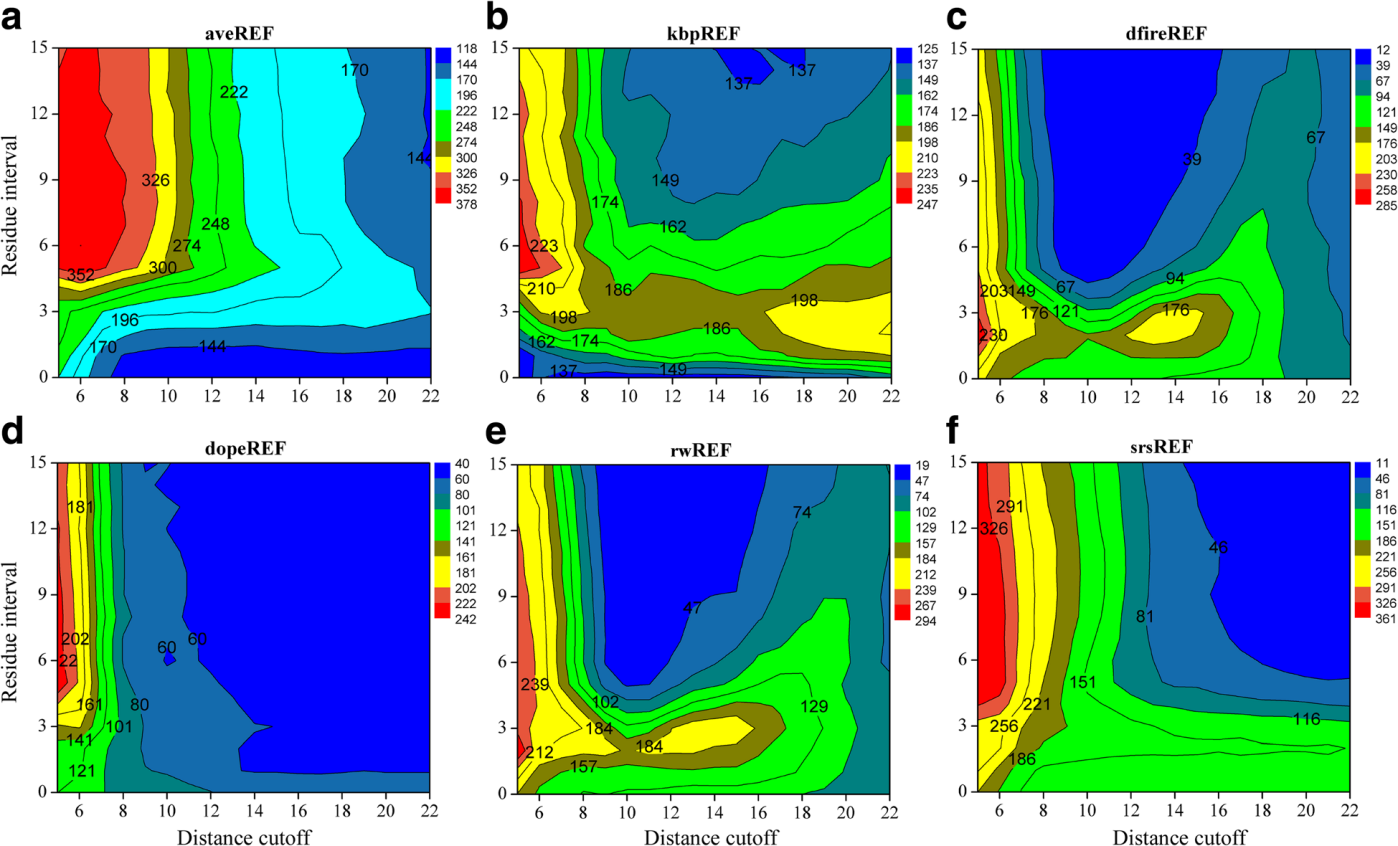
The variation of R1-num with the distance cutoff and residue interval for potentials based on different reference states. R1-num refers to the number of decoy sets whose native structure is given the lowest energy score by the potential. **a**. aveREF. **b**. kpbREF. **c**. dfireREF. **d**. dopeREF. **e**. rwREF. **f**. srsREF

Figure 2 shows the variation of R1-num with distance cutoff and residue interval for potentials based on different reference states. Both distance cutoff and residue interval exhibit significant impacts on the value of R1-num that the potential could achieve. Generally, the shorter the distance cutoff, the higher the achieved value of R1-num, and the highest values are all located at the left margin. The effects of the residue intervals are more related with the reference states. For a given distance cutoff of 5, the best residue intervals range from 4 to 15 for aveREF, dopeREF and srsREF, but are about 5 for kbpREF and about 2 for dfireREF and rwREF. Similar variation trends can be observed in the Z-score plot (Additional file 1: Figure S2). Figure 2 also demonstrates that aveREF outperforms other ones in native recognition, as aveREF recognizes 80% of the native structures (378 out of 468) when adopting the best distance cutoff and residue interval. The second-best potential is srsREF, but its performance is much more sensitive to the choices of distance cutoff and residue interval, which caused R1-num values in a range from 11 to 361. The performances of dfireREF and rwREF are quite similar, and the best R1-num values they can achieve are 285 and 294, respectively. The relatively worst performance in native recognition has dopeREF, which is also most sensitive to the choices of distance cutoff and residue interval.

**Fig. 3:**
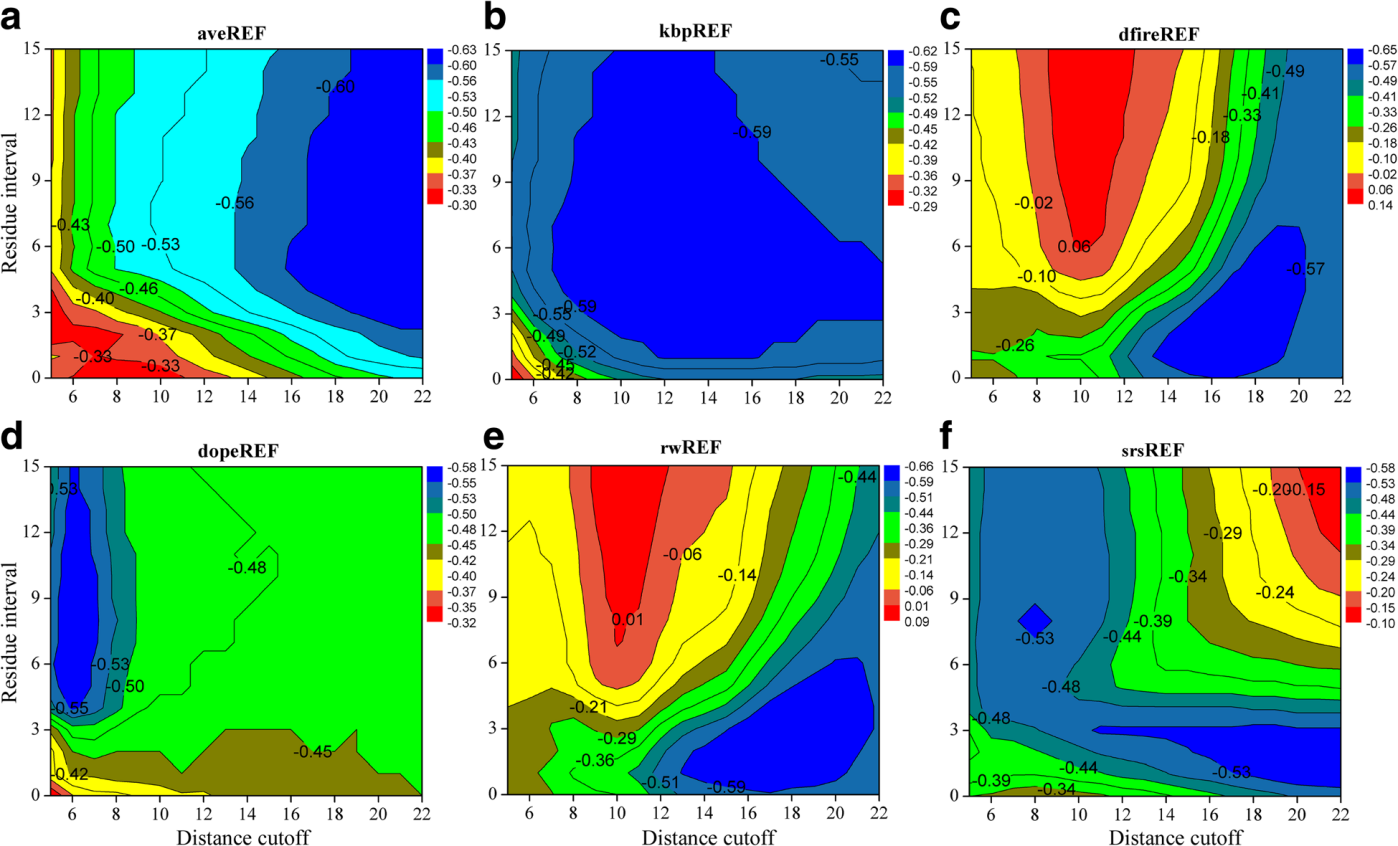
The variation of average PCC between energy score and TM-score with the distance cutoff and residue interval for potentials based on different reference states. PCC refers to Pearson’s correlation coefficient. Since lower energy score (higher TM-score) is desired, the value of PCC is usually negative, the lower the better. **a**. aveREF. **b**. kpbREF. **c**. dfireREF. **d**. dopeREF. **e**. rwREF. **f**. srsREF

Interestingly, the results of decoy discrimination dramatically differ from those of native recognition. As shown in Fig. 3, the best average PCC values (over all 468 decoy sets) between energy score and TM-score (negative value, the lower the better) are located in different regions of the contour figures for potentials based on different reference states. aveREF achieves the best average PCCs when both the distance cutoff and residue interval are relatively large. kbpREF prefers medium values of distance cutoff and residue interval, and its best performance region (the average PCCs are larger than −0.59 except for the four corners of the contour figure) is much broader than potentials based on other reference states. The variation pattern of average PCCs for dfireREF and rwREF is also very similar and resembles that shown in Fig. 2. They are both particularly sensitive to the choices of distance cutoff and residue interval. The best average PCC values they can achieve are −0.65 and −0.66, respectively (by a distance cutoff of about 18 and a residue interval of about 3), but the worst values are around zero (by a distance cutoff of about 10 and a residue interval larger than 6), corresponding to a total inability to distinguish near-native structure from non-native ones. The potential dopeREF achieves the best average PCC values by a distance cutoff of about 6 and a residue interval larger than 4. This is also the only category of potential whose best values of R1-num and average PCC occur in the same region of the contour figure. The potential srsREF shows the best performance by a distance cutoff larger than 16 and a residue interval of about 2. It performs worse when both the distance cutoff and residue interval are relatively larger. In general, the best choices of distance cutoff and residue interval vary sharply with the reference states and measurements. Especially, there is an obvious contradiction in the choice of the distance cutoff to achieve the best R1-num as well as the best average PCC values.

### The potential’s performance on different decoy sets

**Fig. 4:**
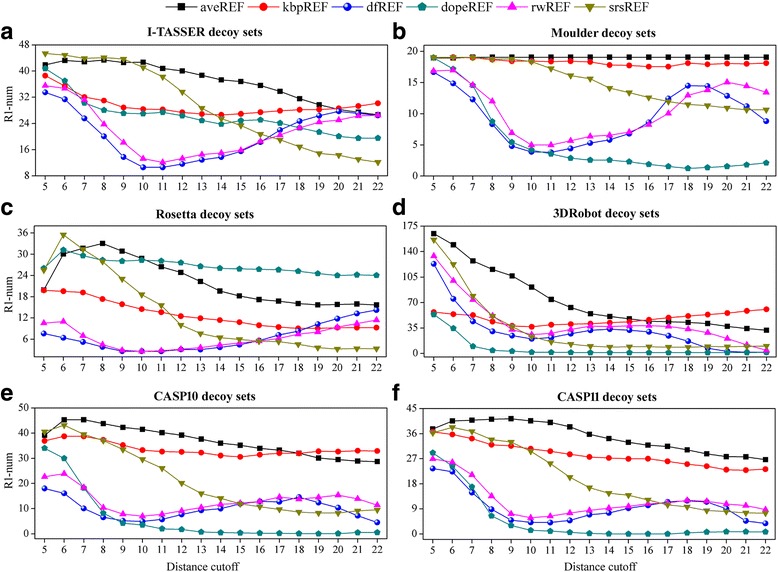
The variation of average R1-num (over all 16 residue intervals) with distance cutoff for the six groups of decoy sets. R1-num refers to the number of decoy sets whose native structure is given the lowest energy score by the potential. **a**. I-TASSER decoy set. **b**. Moulder decoy sets. **c**. Rosetta decoy sets. **d**. 3DRobot decoy sets. **e**. CASP10 decoy sets. **f**. CASP11 decoy sets

**Fig. 5:**
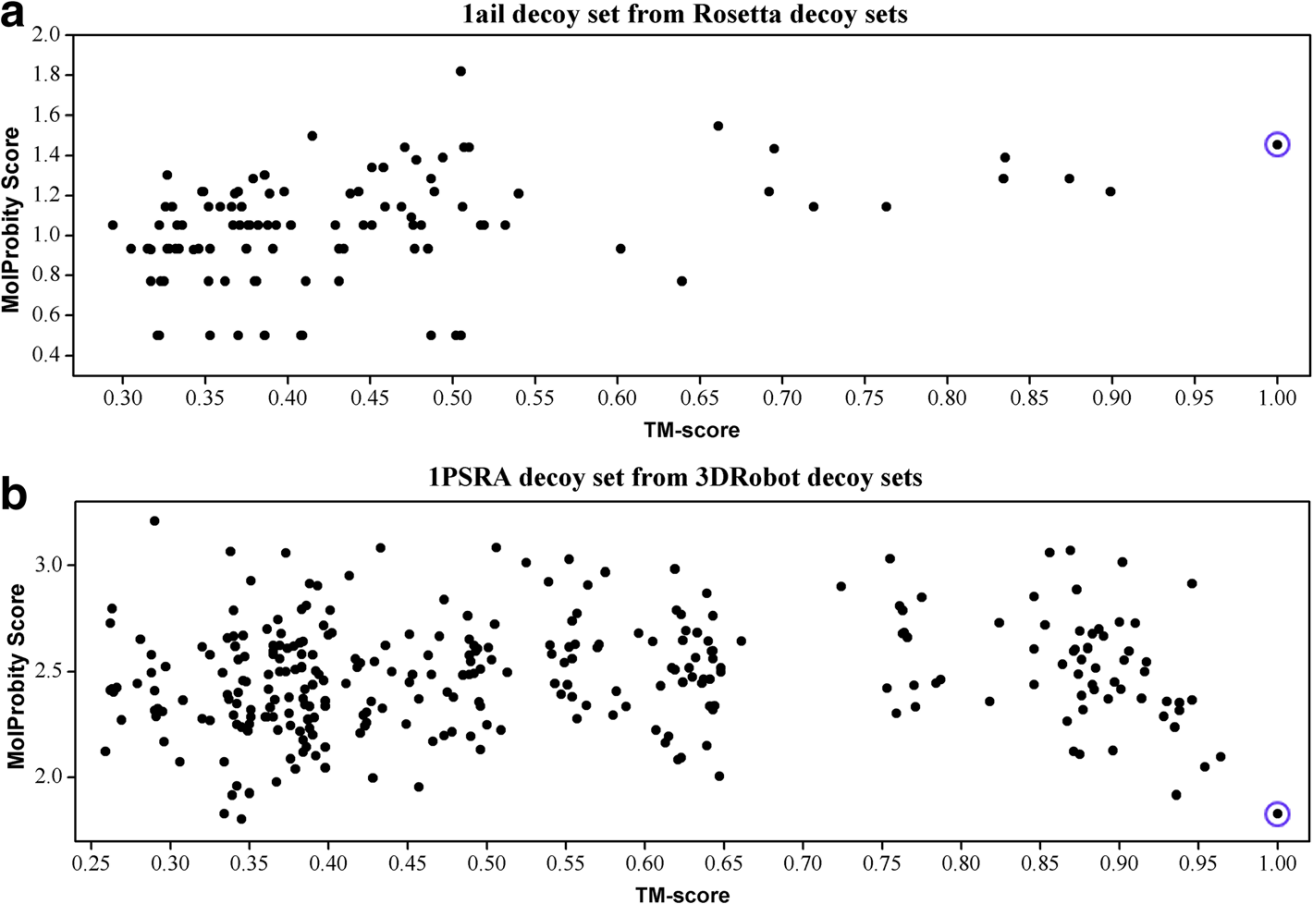
The distribution of MolProbity score from two typical decoy sets. **a** 1ail decoy set from Rosetta decoy sets; **b** 1PSRA decoy set from 3DRbot decoy sets. The native structure is highlighted by open circles

In the above section we demonstrated the general results on all decoy sets. In fact, the best choices of distance cutoff and residue interval vary greatly among different decoy sets, especially when evaluating the ability of native recognition. Figure 4 shows how the average R1-num (over all 16 residue intervals) change with the distance cutoff for the six groups of decoy sets. It is obvious that the highest average R1-num for the I-TASSER, Moulder and 3DRobot decoy sets are all from the potentials with the shortest distance cutoff. However, the distance cutoffs are no longer the shorter the better for the Rosetta and CASP decoy sets. This suggests that the short distance atomic interactions in different decoy sets have different degrees of impact on native recognition. We calculated the MolProbity scores [41] for decoys from the Rosetta and 3DRobot decoy sets (Typical examples are shown in Fig. 5). The results imply that the local structural qualities of decoys from these two decoy sets are at different levels comparing to the qualities of their native structures. The MolProbity scores for decoys from the 3DRobot decoy sets are generally lower than the scores of their native structures, which explains why their short distance atomic interactions (highly related with the local structural qualities) play a more important role in native recognition. On the whole, the distance cutoffs for the best R1-num are commonly in the short side of the given range, which actually means that the inclusion of atomic interactions of larger distances usually introduces more noises than helpful information.

**Fig. 6:**
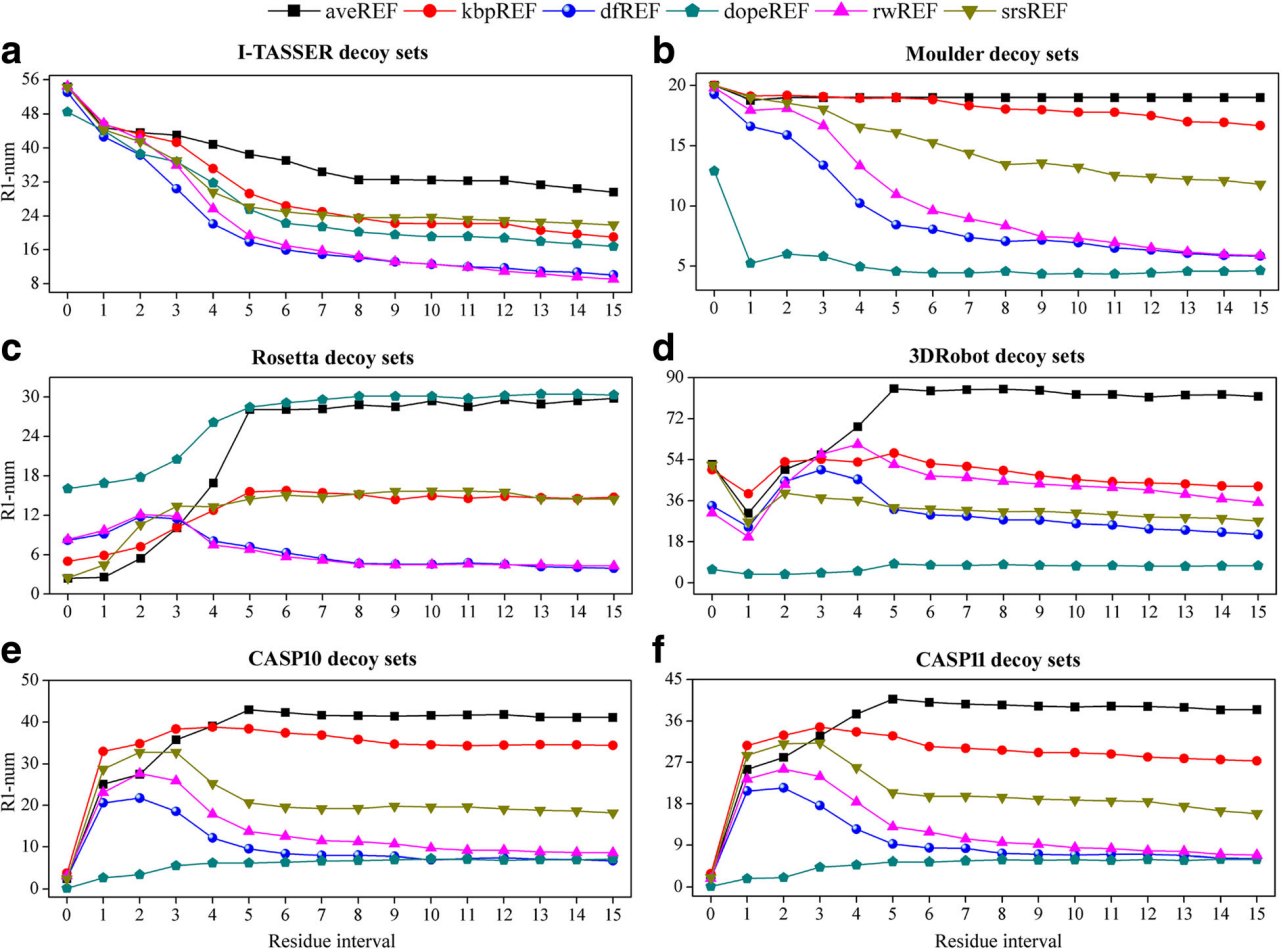
The variation of average R1-num (over all 18 distance cutoff) with residue interval for the six groups of decoy sets. R1-num refers to the number of decoy sets whose native structure is given the lowest energy score by the potential. **a**. I-TASSER decoy set. **b**. Moulder decoy sets. **c**. Rosetta decoy sets. **d**. 3DRobot decoy sets. **e**. CASP10 decoy sets. **f**. CASP11 decoy sets

**Fig. 7:**
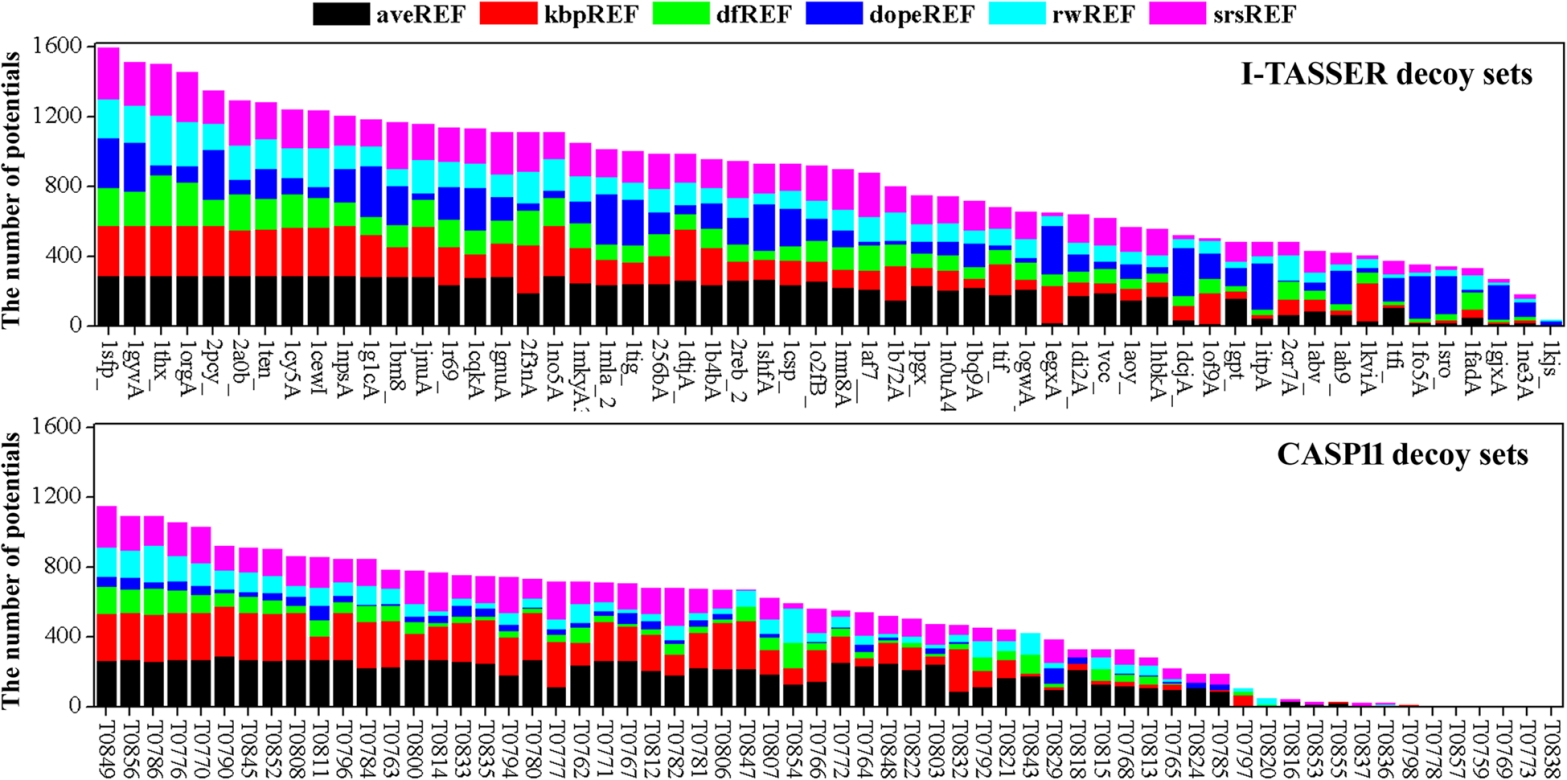
The number of potentials that can recognize the native structure for each set from I-TASSER and CASP11 decoy sets. There are 288 (18 distance cutoffs × 16 residue intervals) potentials on each reference state, and 1728 (288 × 6 reference states) potentials in total

Figure 6 shows how the average R1-num (over all 18 distance cutoffs) vary with residue interval for the different decoy sets. The average R1-num for the I-TASSER and Moulder decoy sets increase rapidly with the decrease of residue interval, and the best performance is achieved by a residue interval of 0. This clearly indicates that the local structure quality (including the conformations of single residues) of decoys from I-TASSER and Moulder is relatively poor, which renders the local atomic interactions especially helpful for telling the native structure apart from decoys. The results of the 3DRobot decoy sets show that the best performance potentials are these with a residue interval of around 4, and the worst performance potentials are those with a residue interval of 1. The performance of potentials with a residue interval of 0 are clearly better than that with a residue interval of 1, which implies that the quality of a single residue of 3DRobot decoys are still somewhat worse than that of the native structures. Along the same line of analysis, the results of the Rosetta and CASP decoy sets suggest that their local structure qualities are pretty good, at least much better than those of the I-TASSER and Moulder decoy sets. Regarding the CASP decoy sets, the inclusion of atomic interactions within single residue greatly weakens the potential’s performance, which implies that decoys with high quality of residue conformation (or side-chain packing) exist in the sets. We used the functional module of residue analysis in MolProbity [41] to perform the residue-by-residue validation on the I-TASSER and CASP11 decoy sets. Additional file 1: Figure S3 shows that the lowest numbers of residue outlier in CASP11 decoys are commonly lower than those of their native structures, while the opposite occurs in I-TASSER decoy sets. In fact, we also estimated the difficulty of a decoy set for native recognition by counting the number of potentials that confer the lowest energy on the native structure. As shown in Fig. 7, the number of potentials that can recognize native structure from I-TASSER decoys are much larger than those from CASP11 decoys. There is no decoy set from I-TASSER whose native structure cannot be recognized, while three native structures from CASP11 sets (T0838, T0773 and T0769) are recognized by no potential and eight native structures can only be recognized by less than 2% of potentials.

**Fig. 8:**
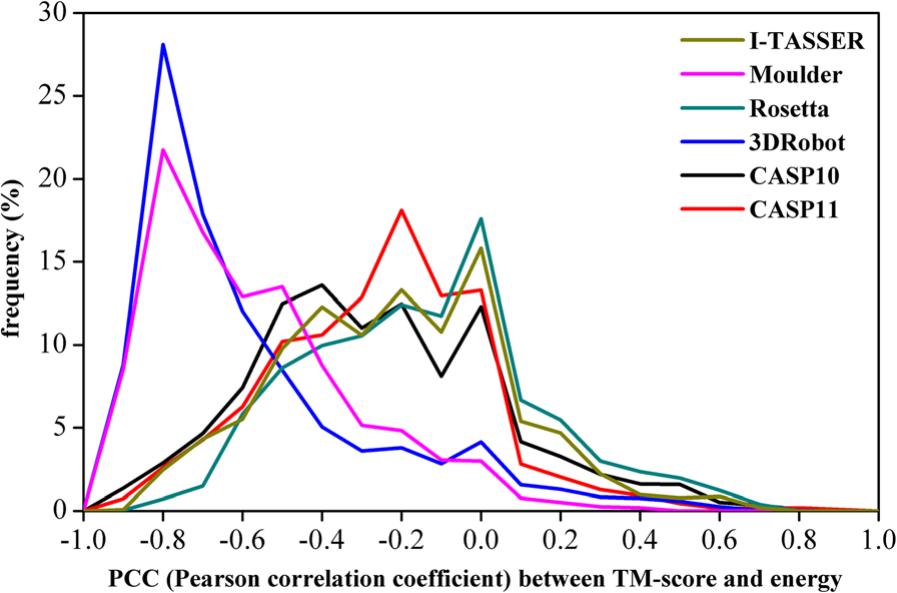
The distribution of PCC between energy score and TM-score from 1728 potentials for the six groups of decoy sets. The bin width of PCC (Pearson’s correlation coefficient) for statistic is 0.1

As shown in Additional file 1: Figure S4, short distance cutoffs are never good choices for potentials to achieve more significant PCCs between energies and TM-score, which is a general observation on all six groups of decoy sets. But the effects of the residue interval vary significantly with different decoy sets (see Additional file 1: Figure S5). For the I-TASSER and Moulder decoy sets, the lower residue intervals yield more significant PCCs, which suggests that decoys with worse backbone structure also have bad local atomic interactions. On the contrary, the local atomic interactions of decoys from the Rosetta and CASP decoy sets do not help discriminate decoys with different backbone qualities. As shown in Fig. 8, the PCCs of the 3DRobot and Moulder decoy sets are much more significant than those of other decoy sets, which is highly related with their great diversity of structural topology.

### Comparison with the existing statistical potentials

Table 3 shows the performance comparisons between the potentials we built and several widely used statistical potentials. Dfire and RW are purely distance-dependent atom-pair potentials, and GOAP is a generalized all-atom statistical potential which includes both distance-dependent and orientation-dependent energy terms. We compared their performances with those of two specific potentials (ave-6-6 and rw-17-3) whose overall performances in native recognition and decoy discrimination are the best respectively. The potential ave.-6-6 successfully recognizes 378 native structures out of 468 decoy sets. This is a significantly larger amount of recognized structures than those the three existing statistical potentials can recognize (134, 123 and 281 respectively). However, the performance of ave.-6-6 in decoy discrimination are clearly worse than those of the existing potentials, especially for the I-TASSER and CASP decoy sets. In contrast, the potential rw-17-3 performs well in decoy discrimination, but relatively poorly in native recognition. Although the overall results of rw-17-3 are better than those of Dfire and RW, it cannot be compared with GOAP. Due to the relatively poor performance on Rosetta and 3DRobot decoy sets, the average PCC of rw-17-3 (−0.66) is slightly weaker than that of GOAP (−0.68).

**Table 3:** Performance comparisons between the potentials we built and several widely-used statistical potentials

Decoy sets	Measurements	Dfire	RW	GOAP	ave-6-6^d^	rw-17-3^e^	Best^f^
I-TASSER	R1-num^a^	43	53	45	42	41	56 (6)
	Z-score^b^	2.80	4.42	4.98	2.42	2.97	11.21 (dope-5-0)
	PCC^c^	−0.47	−0.50	−0.50	−0.09	−0.51	−0.55 (rw-15/16–0)
Moulder	R1-num	18	19	19	19	19	20 (89)
	Z-score	2.67	2.78	3.48	2.97	2.75	8.17 (rw-8-0)
	PCC	−0.84	−0.83	−0.88	−0.52	−0.88	−0.89 (rw-16-2)
Rosetta	R1-num	22	20	45	41	18	48 (ave-8-14, srs-6-8)
	Z-score	1.55	1.48	3.38	3.11	1.46	3.56 (srs-6-7)
	PCC	−0.37	−0.36	−0.51	−0.31	−0.36	−0.45 (srs-6-13/15)
3DRobot	R1-num	1	0	94	176	19	184 (ave-5-5/6)
	Z-score	0.83	−0.30	1.85	3.19	1.16	3.50 (ave-5-5)
	PCC	−0.86	−0.85	−0.90	−0.70	−0.86	−0.88 (ave-19/20/21–5)
CASP10	R1-num	26	16	41	53	31	55 (ave-7-6/7/8)
	Z-score	0.76	0.86	1.60	1.34	1.31	1.70 (dope-6-10/11/12)
	PCC	−0.40	−0.41	−0.53	−0.22	−0.54	−0.56 (rw-18-3, rw-19-4)
CASP11	R1-num	24	15	37	47	33	49 (14)
	Z-score	0.82	1.01	1.91	1.37	1.50	1.72 (dope-6-11)
	PCC	−0.36	−0.40	−0.54	−0.23	−0.52	−0.52 (rw-17-3)
Total/Average	R1-num	134	123	281	378	161	378 (ave-6-6)
	Z-score	1.20	0.95	2.40	2.55	1.55	2.66 (dope-5-5)
	PCC	−0.60	−0.61	−0.68	−0.43	−0.66	−0.66 (rw-17/18–3)

^a^The number of decoy sets whose native structure is given the lowest energy score by the potential

^b^Defined as (<*E*_*decoy*_> − *E*_native_)/*δ*, where *E*_native_is the energy score of native structure, <*E*_*decoy*_> and *δ*are respectively the average and the standard deviation of energy scores of structural decoys

^c^The average Pearson’s correlation coefficient between the energy score and TM-score of all structures in each decoy set, including the native structure

^d^The potential based on the averaging reference state with both distance cutoff and residue interval to be 6

^e^The potential based on the random-walk chain reference state with distance cutoff = 17 and residue interval = 3

^f^The best values among the results of all 1728 potentials with different reference states, distance cutoffs and residue intervals. The corresponding potentials that achieve this values are given in parentheses (e.g. rw-15/16–0 means the potentials rw-15–0 and rw-16-0). Only the number of potentials is given in parentheses if more than 3 potentials can achieve the best value

The last column of Table 3 shows the best results from the 1728 potentials. We can see that the majority of them are much better than those from the existing potentials including GOAP. Nevertheless, for different decoy sets and measurements, the best results are also obtained from different potentials (given in parentheses). All native structures from I-the TASSER and Moulder decoy sets are successfully recognized respectively by 6 and 89 potentials with a residue interval of 0 or 1. The 14 potentials that recognizes 49 native structures from CASP11 decoy sets are all based on the averaging reference state with a distance cutoff around 9 Å and a residue interval from 6 to13.

### Applying the potentials with different residue intervals

**Fig. 9:**
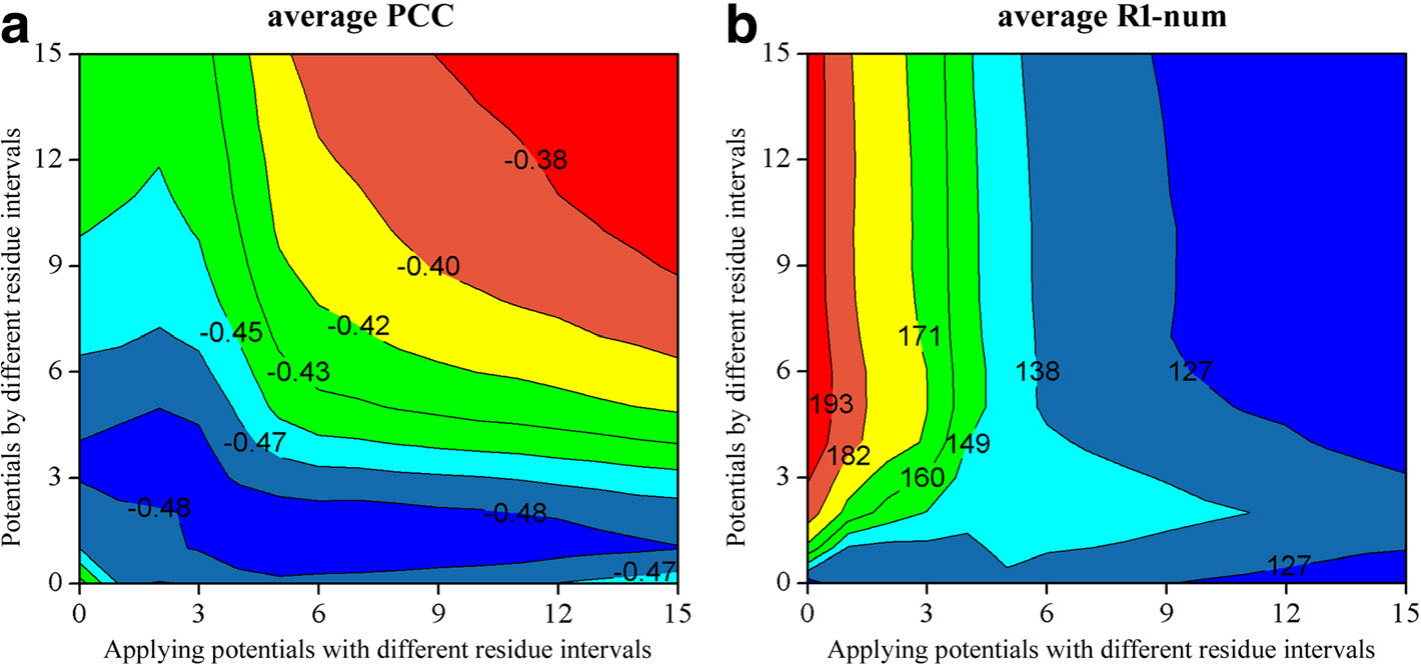
The performance variation when applying potentials with different residue intervals. **a** The variation of average PCC between energy score and TM-score (over six groups of decoy sets and potentials of different distance cutoffs and reference states); **b** The variation of average R1-num (over potentials of different distance cutoffs and reference states)

Generally, the same residue interval is used in both potential construction and application, which does not necessarily represent the best choice. We applied all 1728 potentials by 16 different residue intervals, regardless of what residue interval has been used to construct the potential. Figure 9 shows the results averaged over potentials of different distance cutoffs and reference states. The left panel (Fig. 9a) shows the variation of average PCC between TM-score and potential energies with different residue intervals. For potentials built by low residue intervals (e.g., ≤3), the performances do not vary much when being applied with different residue intervals. However, it is clearly better to adopt lower residue intervals when applying potentials built by higher residue intervals. Figure 9b shows the results of native recognition, which indicates that lower residue intervals are always better than higher ones, no matter by what residue interval the potential has been constructed. These results actually give us a special insight into how the potential’s performance can be improved. However, it should be noted that Fig. 9 shows only the overall results on all potentials and decoy sets, and the performance variation for a specific potential and decoy set may deviate greatly from the overall distribution.

## Conclusions

In this paper, we conducted a comprehensive study on the effects of distance cutoff and residue interval on the performance of distance-dependent atom-pair potential. Hundreds of distance-dependent atom-pair potentials with different distance cutoffs and residue intervals have been constructed based on the same PDB dataset and programming environment. By comparing and analyzing their performances on six groups of decoy sets, we found that the optimal distance cutoff and residue interval are highly related with the reference state that the potential is based on, the measurements of the potential’s performance, and the decoy sets that the potential is applied to. The main findings of this research can be summarized as follows:

- There are no universally optimal distance cutoff and residue interval for potentials based on different reference states.
- The potential’s abilities of native recognition and decoy discrimination cannot be optimized simultaneously with the same distance cutoff. The best distance cutoffs for native recognition are generally shorter than those for decoy discrimination.
- The best choices of distance cutoff and residue interval vary greatly with the specific application environments (decoy spaces). In particular, when the local structural qualities of decoys are evidently inferior to those of the native structures, the potentials with shorter distance cutoff or lower residue interval can usually outperform other potentials.
- Potential’s performance can be further improved by applying the potential with a different residue interval than the one used for potential construction

These conclusions provide basic guidance for the optimization of distance cutoff and residue interval in distance-dependent atom-pair potentials. According to the performance comparisons between the potentials we built and several widely used statistical potentials, the improvements brought by the most suitable distance cutoff and residue interval can enable the distance-dependent atom-pair potentials to outperform many other sophisticated statistical potentials.

## Additional files

Additional file 1: Figure S1. The occurrence frequency of 27,889 atom pairs on the structural dataset of 1762 proteins. **Figure S2.** The variation of Z-score with the distance cutoff and residue interval for potentials based on different reference states. **Figure S3.** The numbers of residue outlier (in native structures and in decoys with the lowest value) for each decoy set from the I-TASSER and CASP11 decoy sets. **Figure S4.** The variation of average PCC between energy score and TM-score (over all 16 residue intervals) with distance cutoff for the 6 bunches of decoy sets. **Figure S5.** The variation of average PCC between energy score and TM-score (over all 18 distance cutoff) with residue interval for the 6 bunches of decoy sets. (DOCX 1096 kb)

Additional file 2: The list of the 1762 non-redundant PDB chains and the detailed information for the filtered CASP11 and CASP12 decoys. (XLSX 339 kb)

## Abbreviations

ave.-6-6: The potential based on the averaging reference state with both distance cutoff and residue interval to be 6
aveREF: Potentials based on averaging reference state
CASP: The Critical Assessment of protein Structure Prediction experiments
dfireREF: Potentials based on finite ideal-gas reference state
dopeREF: Potentials based on spherical non-interacting reference state
kbpREF: Potentials based on quasi-chemical approximation reference state
PCC: The Pearson’s correlation coefficient
rw-17-3: The potential based on the random-walk chain reference state with distance cutoff = 17 and residue interval = 3
rwREF: Potentials based on random-walk chain reference state
srsREF: Potentials based on atom-shuffled reference state
