# Supplementary material for: Diverse effects of distance cutoff and residue interval on the performance of distance-dependent atom-pair potential in protein structure prediction
Source: BMC Bioinformatics. 2017 Dec 8;18:542. doi: 10.1186/s12859-017-1983-3 (PMC5723101; doi:10.1186/s12859-017-1983-3)
Supplement: Supplementary file 1 — The occurrence frequency of 27,889 atom pairs on the structural dataset of 1762 proteins. Figure S2. The variation of Z-score with the distance cutoff and residue interval for potentials based on different reference states. Figure S3. The numbers of residue outlier (in native structures and in decoys with the lowest value) for each decoy set from the I-TASSER and CASP11 decoy sets. Figure S4. The variation of average PCC between energy score and TM-score (over all 16 residue intervals) with distance cutoff for the 6 bunches of decoy sets. Figure S5. The variation of average PCC between energy score and TM-score (over all 18 distance cutoff) with residue interval for the 6 bunches of decoy sets. (DOCX 1096 kb) [file 12859_2017_1983_MOESM1_ESM.docx]

Diverse effects of distance cutoff and residue interval on the performance of distance-dependent atom-pair potential in protein structure prediction

Yuangen Yao^1^, Rong Gui^1^, Quan Liu^1^, Ming Yi^1^, and Haiyou Deng^1,2*^

1 Department of Physics, College of Science, Huazhong Agricultural University, Wuhan, 430070, China

2 Institute of Applied Physics, Huazhong Agricultural University, Wuhan 430070, China

**SUPPLEMENTAL INFORMATION**


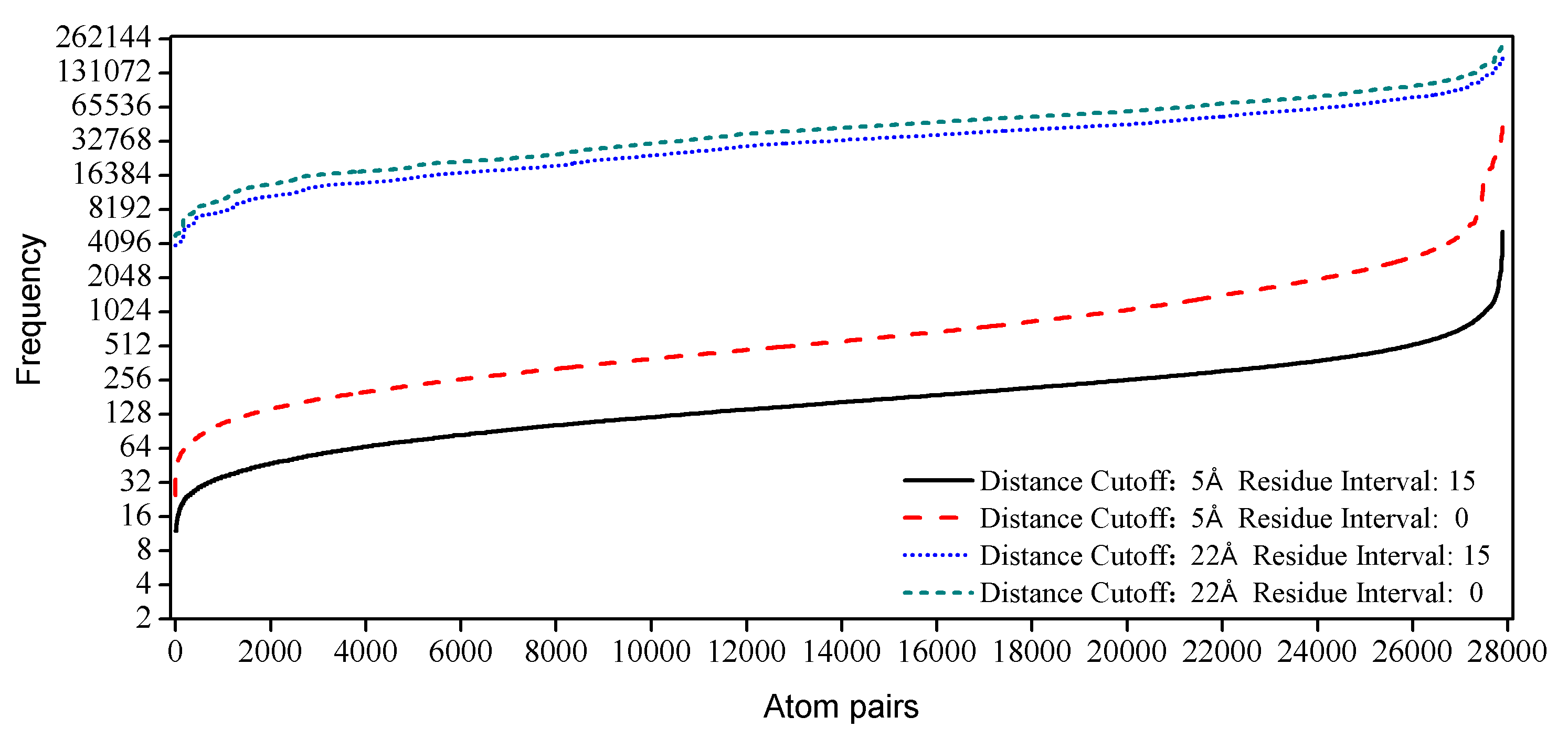


**Figure S1. The occurrence frequency of 27889 atom pairs on the structural dataset of 1762 proteins**

Four cases by different distance cutoff and residue interval are shown. The atom pairs are ordered by their occurrence frequency for each case.


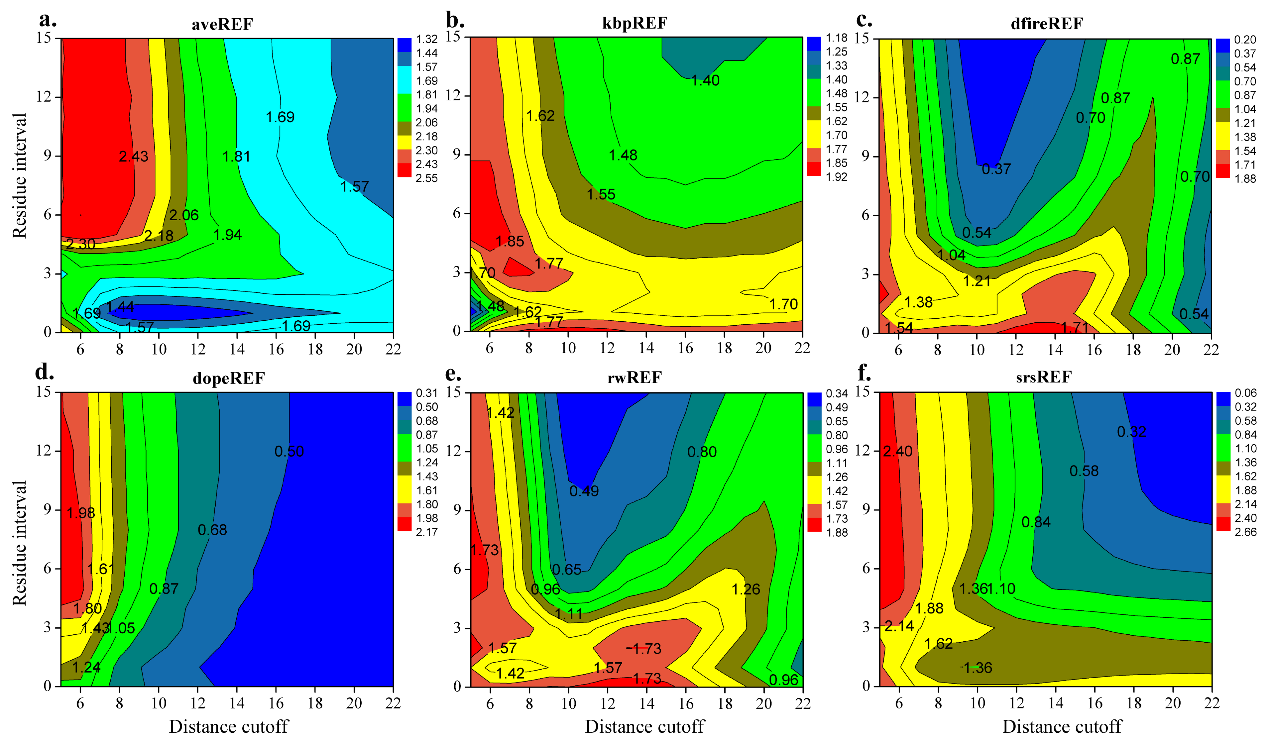


**Figure S2. The variation of Z-score with the distance cutoff and residue interval for potentials based on different reference states.**

**
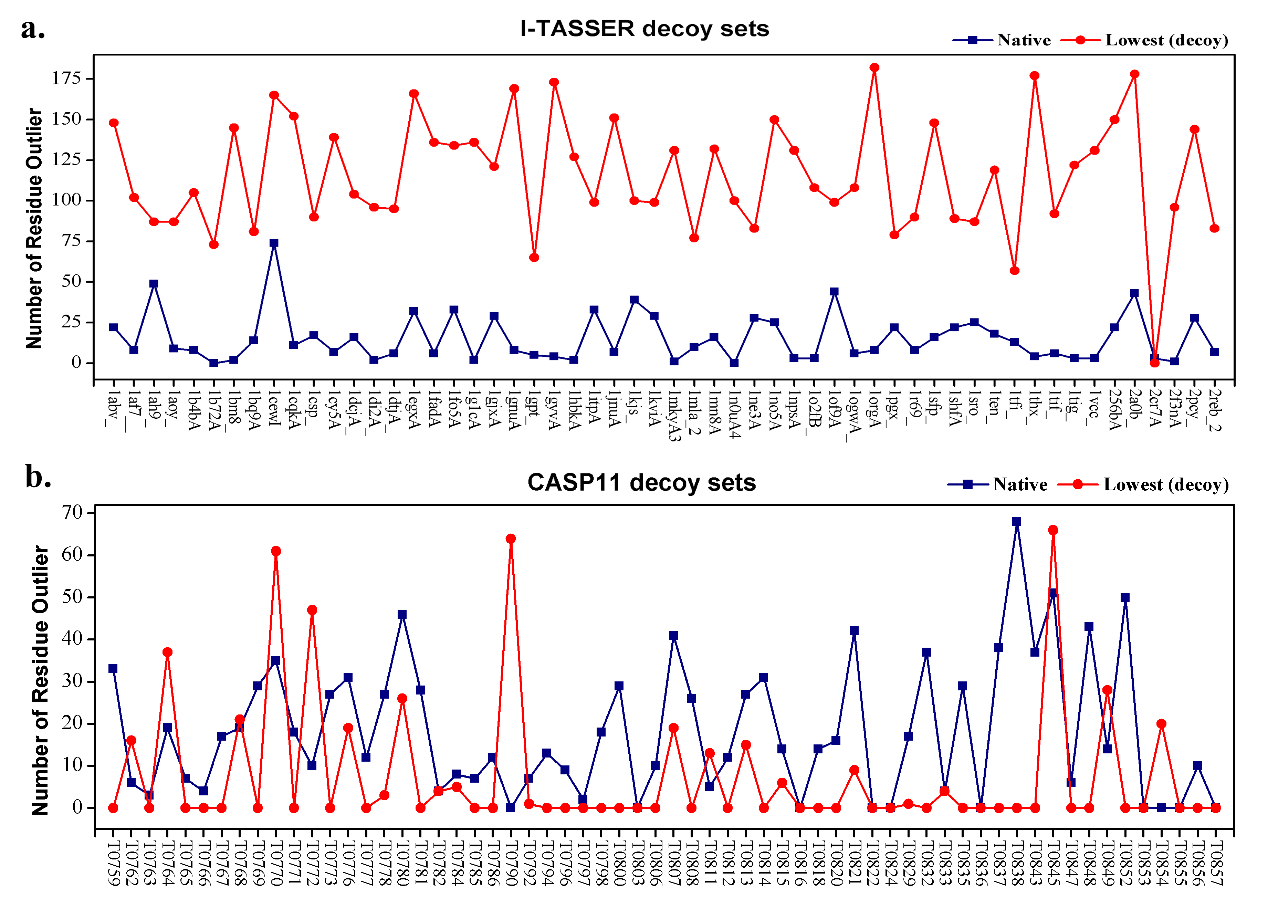
**

**Figure S3. The numbers of residue outlier (in native structures and in decoys with the lowest value) for each decoy set from the I-TASSER and CASP11 decoy sets**

**
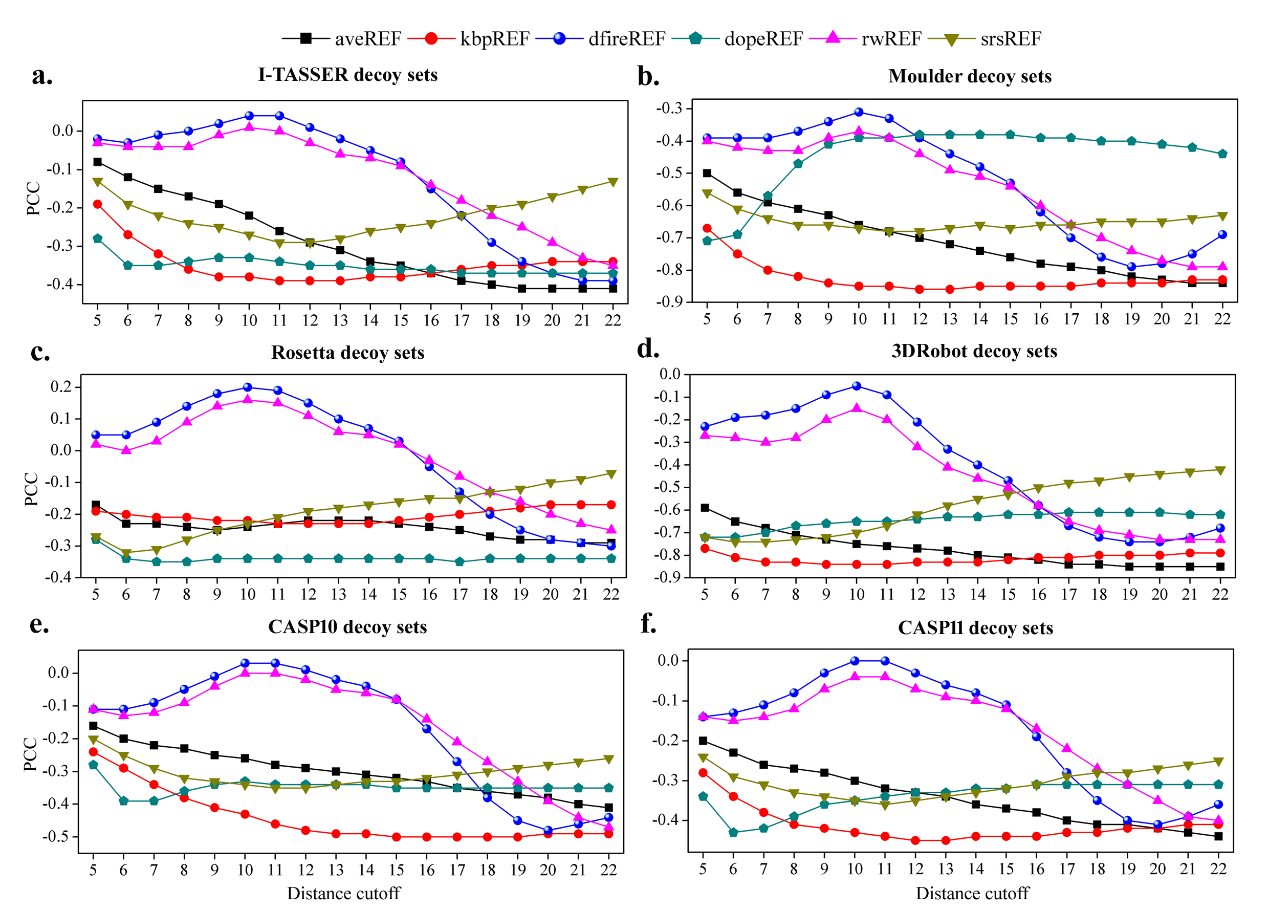
**

**Figure S4. The variation of average PCC between energy score and TM-score (over all 16 residue intervals) with distance cutoff for the six groups of decoy sets**

**
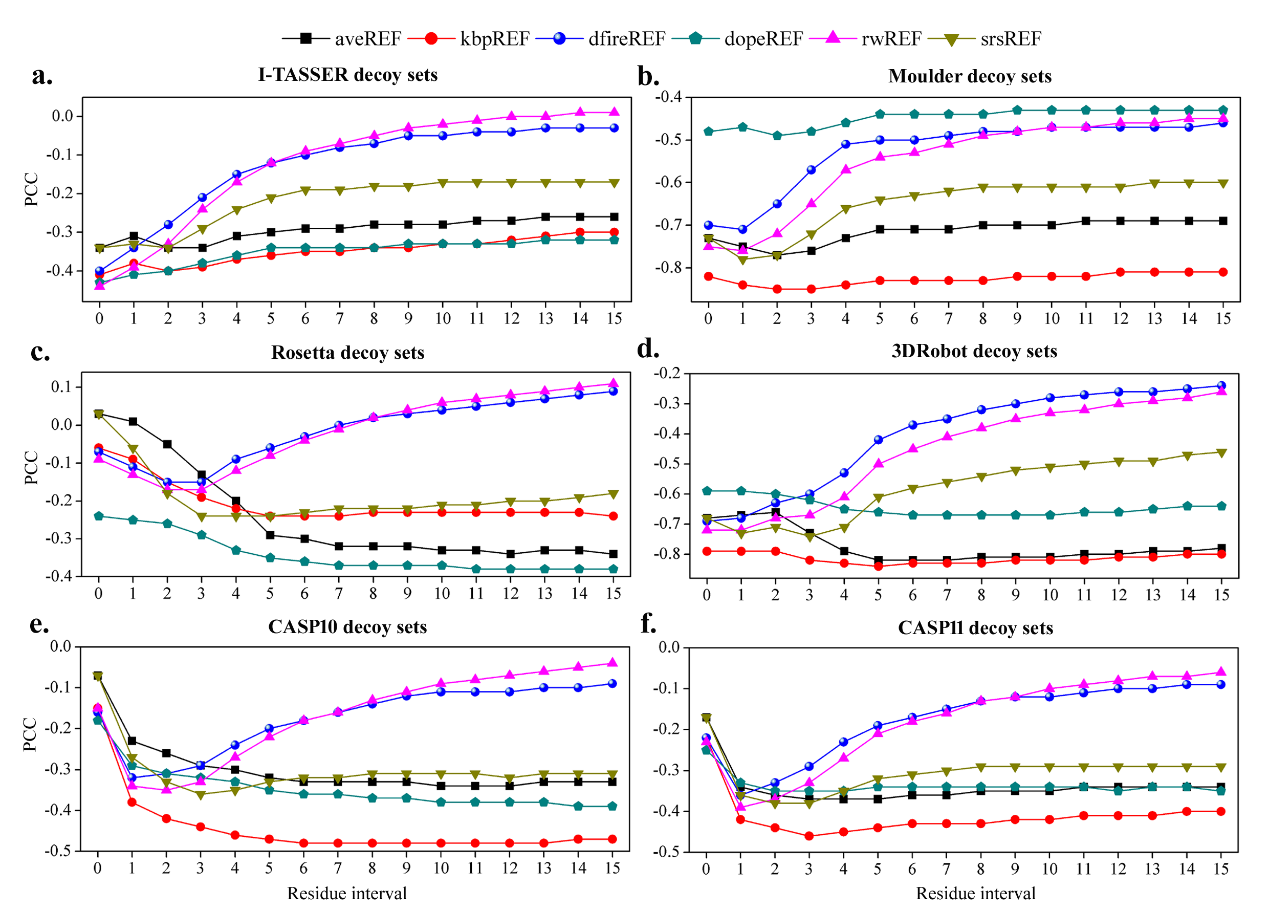
**

**Figure S5. The variation of average PCC between energy score and TM-score (over all 18 distance cutoff) with residue interval for the six groups of decoy sets**
